# Occurrence of respiratory and enteric bacteria and viruses in calves in Iceland

**DOI:** 10.1186/s13028-026-00876-3

**Published:** 2026-07-26

**Authors:** Nicole B. Goecke, Þorbjörg Eva Ellingsen, Lars E. Larsen, Nina D. Otten

**Affiliations:** Department of Veterinary and Animal Sciences, University of Copenhagen, Frederiksberg, Denmark

**Keywords:** Bacteria, Calves, High-throughput qPCR, Iceland, Pathogens, Viruses

## Abstract

**Background:**

Respiratory and gastrointestinal diseases in calves pose a significant challenge to the cattle industry due to their impact on mortality rates, economic losses, animal welfare, and antimicrobial usage. Understanding which pathogens are involved is crucial for optimizing both treatment and preventive measures. In Iceland, bovine respiratory disease in calves is relatively rare, whereas neonatal calf diarrhea is more frequent. However, limited diagnostic resources in the country have resulted in a lack of knowledge about the specific pathogens involved. This study aimed to investigate the occurrence of respiratory and enteric bacteria and viruses in Icelandic calves from birth to three months of age. Twenty (20) herds participated in this cross-sectional study, twelve located in Northeastern (NE) part of Iceland and eight in the Western (W) part, and a total of 197 calves were enrolled in the study. The calves underwent clinical examination, and nasal swab and fecal samples were collected. Herd owners completed a questionnaire regarding calf health, biosecurity, and management practices. The samples were pooled according to regions, seasons, and age groups and analyzed for the presence of selected viruses and bacteria using quantitative real-time PCR.

**Results:**

Among the nasal swab pools, the most identified pathogens were *Trueperella pyogenes* (75%), *Mycoplasma* spp. (45%), *Histophilus somni* (36%), bovine coronavirus (BCoV) (28%), *Pasteurella multocida* (17%), and *Mannheimia haemolytica* (6%). Among the feces pools, BCoV (24%) and rotavirus A (RVA) (23%) were the sole detected pathogens. A greater diversity of pathogens was detected in the NE region than in the W region, since BCoV, *P. multocida* and *M. haemolytica* were not identified in the latter. Diarrhea was the most common clinical sign, and half of the diarrheic calves tested positive for RVA.

**Conclusions:**

This study provides novel insight into the bacterial and viral pathogens circulating in Icelandic calves. Although pathogen diversity was higher in the NE region than in the W region, few clinical signs were observed. Most pathogens were detected across age groups, while seasonal variation in pathogen occurrence was limited in the NE region. These findings improve our understanding of pathogen distribution in Iceland and may support future disease surveillance and preventive strategies.

**Supplementary Information:**

The online version contains supplementary material available at https://doi.org/10.1186/s13028-026-00876-3.

## Background

Respiratory and gastrointestinal diseases in calves represent a major challenge to the cattle industry, with significant implications for mortality, animal welfare, productivity, antimicrobial usage, and economic losses for producers. The two predominant disease complexes affecting calves are neonatal calf diarrhea and bovine respiratory disease (BRD), both of which are multifactorial in nature. These conditions are influenced by a combination of infectious agents, environmental stressors, and the health and immunological status of the animal [1, 2].

Neonatal calf diarrhea occurs primarily in calves aged 0–4 weeks and is associated with a range of enteric pathogens including bovine coronavirus (BCoV), rotavirus A (RVA), bovine viral diarrhea virus (BVDV), *Escherichia coli* F5 (K99+), *Salmonella enterica serovar Dublin*, *Clostridium perfringens*, *Cryptosporidium parvum*, and *Eimeria* spp. Many of these pathogens are age-specific in their association with disease [3–5]. BRD, while affecting cattle of all ages, tends to be more severe in younger calves and involves a wide array of viral agents such as bovine respiratory syncytial virus (BRSV), BCoV, bovine parainfluenza virus type 3 (BPiV3), and influenza D virus (IDV), as well as bacterial agents including *Mannheimia haemolytica*, *Pasteurella multocida*, *Histophilus somni*, and *Mycoplasma bovis* [6–9]. Opportunistic bacteria, including *Trueperella pyogenes* and less pathogenic *Mycoplasma* species (spp.), have occasionally been associated with chronic cases of BRD complex [10, 11]. Bovine herpesvirus 1 (BHV-1) and BVDV may also contribute to BRD, although these viruses have been eradicated in several countries, and have never been detected in Iceland. While the involvement of pathogens in neonatal calf diarrhea and BRD is well documented in many countries, these findings may not be directly applicable to Iceland due to its geographical isolation and strict regulations on livestock importation. Iceland is placed in the North Atlantic, just south of the Arctic Circle between Greenland and Europe. There are around 452 dairy farms in Iceland with varying size and number of cows and calves. It is estimated that Iceland has around 75,000–80,000 cattle, of which approximately 26,000 are dairy cows, and the remainder are calves and young animals [12].

Limited data have been published regarding the presence of pathogens in Icelandic calves. One study has reported detection of *Eimeria* oocysts in all tested calves from three herds, of which 55% exhibited diarrhea [13]. *T. pyogenes*, *P. multocida*, and *M. haemolytica* have previously been identified in Icelandic cattle (personal communication, Vilhjálmur Svansson, Institute for Experimental Pathology at Keldur, University of Iceland). In 2022, a dairy farm located in northeastern Iceland experienced an outbreak of diarrhea among cows, accompanied by dry coughing and occasional epistaxis. Serological testing revealed the presence of antibodies against BPi3, marking the first documented case of this virus in Iceland [14]. BCoV is endemic in the country and is associated with annual reductions in milk yield (personal communication, Vilhjálmur Svansson, Institute for Experimental Pathology at Keldur, University of Iceland). Icelandic cattle are genetically distinct from other Western European breeds, with close genetic ties to three Finncattle breeds (Eastern, Northern and Western Finncattle) as well as Swedish Mountain cattle [15].

Despite the high prevalence of infectious diseases in cattle production, laboratory diagnostics remain underutilized in many countries, primarily due to cost and logistical challenges related to sample collection and analysis. Identifying the causative agents of disease is essential for effective prevention and treatment strategies. The pathogens associated with neonatal calf diarrhea and BRD are capable of causing disease independently, but they frequently occur in mixed infections, which tend to result in more severe clinical outcomes [16–18]. Therefore, it is preferable to be able to test for multiple pathogens in a single setup, which can be achieved using previously described high-throughput quantitative real-time PCR (qPCR) and multiplex qPCR systems [19–22].

Little is known about the pathogens circulating among Icelandic calves, as limited research has been conducted in this area. Therefore, the aim of this study was to investigate the occurrence of respiratory and enteric bacteria and viruses in Icelandic calves from birth to three months of age. Furthermore, the study sought to explore potential trends in pathogen occurrence across geographical regions, and age.

## Methods

### Herd description and sample collection

Nasal swab and fecal samples were collected from 20 cattle herds, where 12 herds were located in the Northeastern (NE) region and eight herds were located in the Western (W) region of Iceland. Sampling was done in the NE region in April 2023 (referred to as spring) and September 2023 (referred to as fall) in the same 12 herds, while sampling was only performed in September 2023 in the W region. This convenience sampling of herds was chosen due to limitations in available time and financial resources. The samples were collected from calves in three age groups: group 1 (0–10 days), group 2 (14–28 days) and group 3 (90–110 days). Due to the relatively small herd sizes in Iceland (in average 54.4 cows per herd [23]), herds were selected based on herd size to ensure a sufficient number of calves per herd, since an inclusion criterion was to have at least one calf in at least two of the three age groups. If more than three calves were present in an age group, three calves were selected by the investigator from a list of eligible calves provided by the farmer. Thus, a non-random convenience sample of eligible calves was obtained. The initial plan was to collect samples from three calves per age group in each herd, but this was not always possible due to an insufficient number of calves in the age group. In total, 197 calves (66 calves aged 0–10 days, 71 calves aged 14–28 days and 60 calves aged 90–110 days) from the 20 herds were examined. Nasal swab samples were collected from 196 of the calves, while feces samples were collected from 193 of the animals (Additional file 1).

The nasal swab samples were collected by inserting a sterile cotton swab approximately 8–10 cm into both nares and turning the swab around for a few seconds, and immediately after, the swab was placed and stored in 1 mL of liquid amies medium (ESwab^®^ 480 C, Copan, Murrieta, CA). Fecal samples were collected from the rectum of the calves by stimulation of rectal evacuation movements using a gloved finger and the expelled feces were collected in a sterile cup. The samples were kept cold (ཞ5°C) right after collection and were subsequently stored at −20 °C. All samples were shipped frozen to the University of Copenhagen (Frederiksberg, Denmark), where the samples were stored at −80 °C until further analysis.

All the participating farmers were asked to complete a questionnaire regarding biosecurity, calf health, colostrum management and other management practices at the sampling visit (Additional file 2).

## Clinical examination

At each sampling, clinical signs (ocular and nasal discharge, ear and head tilt, coughing, body temperature, consistency of feces) were registered for each sampled calf and scored based on the scoring system described previously [24]. For each sign, clinical scores were rated depending on the severity, and a score of zero was given if the sign was not present. For coughing, the lowest score (zero) was given if no coughs were observed and the highest score (three) if repeated unprovoked coughs were observed. For nasal and ocular discharge, clear serous discharge equaled a score of one, while mucopurulent discharge equaled a score of three. For ear and head tilt, the highest score (three) was given if bilateral ear drop was present or head was tilted. For feces, the lowest score (zero) was given if the feces content was semi-formed and pasty, score one was given for loose and not-formed feces and score two was given if feces was watery, heavy mucus and/or bloody. Body temperature was measured rectally and categorized as either normal at temperatures equal to or below 39.4 °C, or as febrile when exceeding this cut-off.

### Nucleic acid extraction

Nasal swab samples and fecal samples were pooled according to age, season, and herd. The pools consisted of one to four individual samples. In addition, fecal samples collected from calves with a fecal score of two (i.e. watery/heavy mucus/bloody) were also analyzed individually.

Prior to nucleic acid extraction, each nasal swab sample was prepared by vortexing to transfer the biological material into phosphate-buffered saline (PBS), followed by centrifugation for 3 min at 5,500 *x* g. The nasal swab samples were pooled using equal volumes (µL) from each individual sample, and 200 µL of the pooled sample was used for extraction. For each fecal sample, a 10% dilution in PBS was prepared by weighing 0.1 g of feces and adding PBS. One 5-mm steel bead (QIAGEN, Hilden, Germany) was added to each sample, and the sample was homogenized in a TissueLyser II (QIAGEN) for 20 s at 15 Hz. The homogenate was centrifuged for 90 s at 6,700 *x* g. The 10% fecal dilutions were pooled using equal volumes (µL) from each individual sample, and 200 µL of the pooled sample was used for extraction. RNA and DNA were extracted using the extraction robot QIAcube HT (QIAGEN) and the IndiSpin QIAcube HT pathogen kit (Indical Bioscience, Leipzig, Germany) according to the manufacturer’s instructions. Positive and negative (nuclease-free water) controls were included in each extraction. The nucleic acids were stored at −80 °C until further analysis.

### High-throughput real-time PCR analysis

Prior to the high-throughput qPCR analysis, the extracted samples were run through a reverse transcription and/or pre-amplification step as described in Goecke et al. 2021 [19]. For the high-throughput qPCR analysis, the high-throughput qPCR platform BioMark HD (Standard BioTools, San Francisco, CA) and the 192.24 dynamic array (DA) integrated fluidic circuit (IFC) nanofluidic chip (Standard BioTools) were used. The 192.24 DA IFC combines 192 samples with 24 assays for 4,608 individual and simultaneous qPCR reactions.

A 4 µL sample mix was prepared for each of the samples by mixing 2.2 µL pre-sample mix (prepared by mixing 2 µL of 2X TaqMan Gene Expression Mastermix (Applied Biosystem, Foster City, CA) and 0.2 µL of 20X sample loading reagent (Standard BioTools) with 1.8 µL of the pre-amplified sample. Assay mix for each PCR assay was made by mixing 2 µL primer/probe stock (containing 33 µM of each primer and 10 µM of probe) with 2 µL of 2X assay loading reagent (Standard BioTools). Three (3) µL of assay mix and 3 µL of sample mix was loaded into the respective inlets of the 192.24 DA IFC chip. The 192.24 DA IFC chip was placed in the IFC controller RX for loading and mixing for approximately 30 min. Finally, the chip was inserted into the high-throughput qPCR platform BioMark HD (Standard BioTools) and analyzed with the following thermal cycle conditions: 50 °C for 2 min, 95 °C for 10 min followed by 40 cycles of 95 °C for 15 s and 60 °C for 60 s. In each chip run, positive and non-template (UltraPure™ DNase/RNase-Free Distilled Water; Thermo Fisher Scientific) controls were included. Amplification curves and quantification cycle (Cq) values were obtained on the BioMark HD system and finally analyzed using Fluidigm Real-Time PCR Analysis software 4.8.1 (Standard BioTools).

The samples were analyzed for the presence of 11 different bacteria and viruses known to be able to cause either respiratory or gastrointestinal diseases in calves: BPiV3, BRSV, IDV, RVA, *M. haemolytica*, *P. multocida*, *H. somni*, *Mycoplasma* spp., *M. bovis*, *T. pyogenes* and *E. coli* F5. The used primer and probe sequences have been published elsewhere [19] except for BPi3 where the following oligonucleotides were used: BPiV3-F; GAGTTYGCACCAGGYAAYTATC, BPiV3-R; ACTGCTTGACCYAGTTGGAAC and BPiV3-P; FAM-TGGAGTTATGCRATGGGTGTAGCAGTTGT-BHQ1. For the *Mycoplasma* spp. assay, a modified version of the probe (FAM-TGTGTGCCTAATACATGCATGTYGAGCGA-BHQ1) was used.

### BCoV real-time RT-PCR analysis

The samples were also tested for the presence of BCoV by RT-qPCR using primer (Fw: GTTGGTGGAGTTTCAACCCAG and Rv: TGACCACGTATTATTGTGACCGT) and probe (FAM-TGGTAGTCCTCAATTATMGGCCTAACAT-BHQ1) sequences modified from [19]. The RT-qPCR assay was performed in a final volume of 15 µL using AgPath-ID™ one-step RT-PCR reagents kit (Applied Biosystems) with 2 µL RNA. The PCR mix consisted of 7.5 µL 2X RT-PCR buffer, 0.5 µL of each primer (10 µM), 0.5 µL probe (10 µM), 0.6 µl 25X RT-PCR enzyme mix and 3.4 µL nuclease-free water. The qPCR reactions were run at the following thermal cycle conditions: 45 °C for 20 min, 95 °C for 10 min followed by 45 cycles of 94 °C for 15 s and 60 °C for 45 s. The RT-qPCR assay was run on the Rotor-Gene Q platform (QIAGEN), and data, including Cq values, were analyzed using Rotor-Gene Q software version 2.3.5 (QIAGEN). In all the RT-qPCR runs, positive and non-template (UltraPure™ DNase/RNase-Free Distilled Water; Thermo Fisher Scientific) controls were included.

### Statistical analysis

All data management and statistical evaluations of pathogen presence in the various age groups, regions and seasons were performed using the statistical software R (version 4.3.2) (R Core Team, 2025). Since the outcome variables were created by pathogen presence in pooled nasal swab and fecal samples for either age group in each herd, the overall sample was reduced to 83 pools for each sample type. Additionally, the presence of pathogens in both sample types was limited, so data were assumed to be non-gaussian. Associations between pathogen presence and explanatory variables age group (1, 2, 3) and regions (NE, W) were evaluated by means of Fisher´s Exact test. Assessment of seasonal difference from the 12 herds in region NE, where data were clustered within herds and repeated across season. Hence, the probability of pathogen presence between seasons and across age groups was modelled, using a hierarchical generalized linear mixed model (GLMM), and repeated testing within herds was accounted for by including herd as random effect by using the *glmer* function. All significant associations were assessed at a significance level of *P* = 0.05.

## Results

### Overall pathogen occurrence

Nasal swab samples (*n* = 196), fecal samples (*n* = 193) and registration of clinical signs (*n* = 197) were collected from Icelandic calves within three age groups (0–10 days, 14–28 days, 90–110 days) derived from 20 different herds distributed in two regions (NE and W). The number of cows per herd varied from < 40 to > 120 with most herds having a size of 40–80 cows (Table 1 and Additional file 1).

**Table 1:** Number of herds and calves in the NE and W regions in three age groups

Age groups				1	2	3
		Herds (*n*)	Calves (*n*)	0–10 d	14–28 d	90–110 d
**Total number**		20	197	66	71	60
**Region** (season)	NE (spring)	12	69	25	25	19
	NE (fall)		86	26	31	29
	W (fall)	8	42	15	15	12
**Herd size** (no. of cows)	< 40	2	8	2	5	1
	40–80	10	104	30	38	36
	81–120	6	64	24	20	20
	> 120	2	20	9	8	3

In total, 83 nasal swab pools (1–3 samples per pool) were analyzed, and *T. pyogenes* (75%), *Mycoplasma* spp. (45%), *H. somni* (36%), were found to be the most prevalent pathogens across all age groups followed by BCoV (28%), *P. multocida* (17%) and *M. haemolytica* (6%), while BRSV, BPi3, IDV and *M. bovis* were not detected in any of the pools. *T. pyogenes*, *Mycoplasma* spp. and *H. somni* were detected in all three age groups in both regions (NE and W) and seasons (spring and fall), while BCoV (28%), *P. multocida* (17%) and *M. haemolytica* (6%) were only detected in NE and the latter was only found to be present in age group 3 (90–110 days) (Table 2). Additionally, significant differences in presence of *T. pyogenes* were found between regions (*P* = 0.001), with a higher prevalence of positive pools in region NE. The only significant difference in pathogen occurrence between age groups was found for *M. haemolytica* (*P* = 0.002), which was detected in two herds in both spring and fall, whereas in a third herd it was only detected in spring.

**Table 2:** Occurrence of respiratory pathogens in pooled nasal swab samples by region, season, and age group. The number of positive pools for each pathogen is shown in parentheses

Region	Season	Age	No. calves (no. pools)	Mycoplasma. spp	M. haemolytica	H. somni	*P*. multocida	T. pyogenes	BCoV
**NE**	Spring	1	25 (11)	55% (6)	0% (0)	45% (5)	9% (1)	91% (10)	36% (4)
**NE**	Spring	2	25 (11)	27% (3)	0% (0)	27% (3)	18% (2)	73% (8)	64% (7)
**NE**	Spring	3	19 (9)	33% (3)	33% (3)	33% (3)	33% (3)	78% (7)	0% (0)
**NE**	Fall	1	26 (11)	55% (6)	0% (0)	27% (3)	18% (2)	64% (7)	36% (4)
**NE**	Fall	2	31 (11)	64% (7)	0% (0)	45% (5)	27% (3)	82% (9)	36% (4)
**NE**	Fall	3	28 (11)	45% (5)	18% (2)	45% (5)	27% (3)	91% (10)	36% (4)
**W**	Fall	1	15 (7)	43% (3)	0% (0)	29% (2)	0% (0)	57% (4)	0% (0)
**W**	Fall	2	15 (6)	33% (2)	0% (0)	33% (2)	0% (0)	67% (4)	0% (0)
**W**	Fall	3	12 (6)	33% (2)	0% (0)	33% (2)	0% (0)	50% (3)	0% (0)
**Total**			196 (83)	45% (37)	6% (5)	36% (30)	17% (14)	75% (62)	28% (23)

**Fig. 1:**
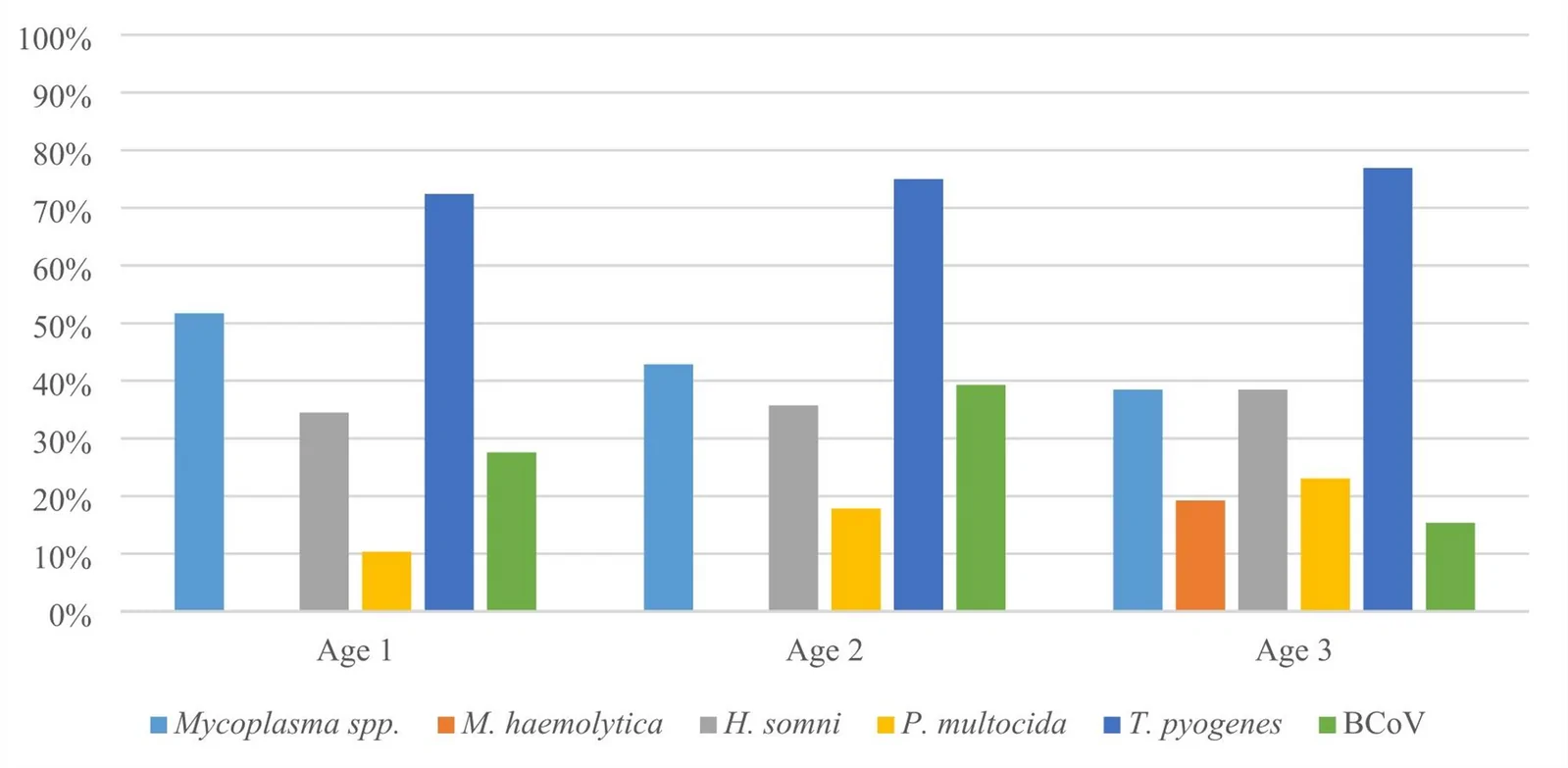
Occurrence of respiratory pathogens in the three age groups across herds, regions, and seasons

Distribution of the respiratory pathogens in the three age groups across the herds, regions and seasons, showed that the occurrence of *T. pyogenes*, *P. multocida* and *H. somni* increased with age, while it decreased for *Mycoplasma* spp. The highest occurrence of BCoV was observed in age group 2 (Fig. 1).

In the NE region, the 12 herds were sampled both in the spring and in the fall. The same respiratory pathogens were found to be present in both seasons although with slightly different prevalences. Across both seasons, presence of *T. pyogenes* was numerically most dominant (spring–fall: 81%–79%) followed by *Mycoplasma* spp. (39%–55%), *H. somni* (35%–39%), BCoV (35%–36%), *P. multocida* (19%–24%) and *M. haemolytica* (10%–6%), however, none of the differences in pathogen presence between seasons were statistically significant.

Only a few calves exhibited clinical signs of respiratory disease at the time of sampling. A total of 1.5% of the calves (3/197) scored two or higher in two or more clinical categories (Additional file 1). Of these, two calves originated from region NE and one from region W. They were of different age groups, and all showed signs of disease during the fall. Coughing was observed in 5% of the calves, either provoked (*n* = 8, score 1) or spontaneous (*n* = 1, score 2). Minor neurological signs, such as ear and head tilt, were present in 1% of the calves (*n* = 1, score 1; *n* = 1, score 2). Transparent ocular discharge was recorded in 2% of the calves (*n* = 4, score 1), and nasal discharge in another 2% (*n* = 2, score 1; *n* = 2, score 2). Additionally, 12% of the calves had a rectal temperature exceeding 39.4 °C (*n* = 24) (Additional file 1).

In total, 83 fecal pools (1–3 calves per pool) were analyzed and RVA was detected in 23% of the pools, while BCoV was detected in 24% of the pools. *E. coli* F5 were not detected in any of the pools. The highest occurrence of RVA was found in age group 1 (0–10 days) and a decreasing incidence with age was observed for both regions (NE and W) and in both seasons in the NE region. This difference in age distribution was significant (*P* = 0.001). BCoV occurrence was significantly higher across all age groups in the NE region (*P* = 0.004), while no calves tested positive in the W region (Table 3).

**Table 3:** Occurrence of enteric pathogens in pooled fecal samples by region, season, and age group. The number of positive pools for each pathogen is shown in parentheses

Region	Season	Age Group	No. calves (no. pools)	RVA	BCoV
**NE**	Spring	1	24 (11)	55% (6)	36% (4)
**NE**	Spring	2	25 (11)	27% (3)	55% (6)
**NE**	Spring	3	19 (9)	0% (0)	11% (1)
**NE**	Fall	1	26 (11)	55% (6)	27% (3)
**NE**	Fall	2	31 (11)	9% (1)	36% (4)
**NE**	Fall	3	26 (11)	0% (0)	18% (2)
**W**	Fall	1	15 (7)	29% (2)	0% (0)
**W**	Fall	2	15 (6)	17% (1)	0% (0)
**W**	Fall	3	12 (6)	0% (0)	0% (0)
**Total**			193 (83)	23% (19)	24% (20)

Of the tested calves, 8% exhibited clinical signs of diarrhea (*n* = 16, score 2, i.e. watery/heavy mucus/bloody), while 15% of the calves had loose and not-formed feces (*n* = 29, score 1). Of the calves with diarrhea, 12% of them belong to age group 1, while 8% and 4% belong to age group 2 and 3, respectively (Additional file 1). Feces samples from the diarrheic calves were also tested as individual samples and here 50% of the samples tested positive for RVA – off with 75% of these derived from the youngest calves (age group 1). BCoV was detected in 25% of the individual samples with two calves in age group 1 and 2, respectively, testing positive.

No detectable seasonal shift in pathogen circulation among these herds was found as shown in the results from the GLMM in Table 4. Only six pathogens displayed enough variation to successfully run the models. Across these pathogens, odds ratios ranged from 0.69 to 1.96, and all 95% confidence intervals included 1, indicating no evidence of increased pathogen detection in either spring or fall for the herds in the NE region.

**Table 4:** Summary of the GLMM evaluating seasonal differences in pathogen occurrence within the NE region

Pathogen	Sample Type	Odds ratio	Lower CI	Upper CI	*P* value
***P. multocida***	Nasal swab	1.27	0.249	6.51	0.772
***H. somni***	Nasal swab	1.19	0.419	3.39	0.743
***Mycoplasma*** **spp**.	Nasal swab	1.96	0.711	5.39	0.193
***T. pyogenes***	Nasal swab	0.849	0.242	2.98	0.798
**BCoV**	Nasal swab	1.12	0.343	3.69	0.846
**BCoV**	Fecal	0.692	0.226	2.11	0.518

### Questionnaire

In the NE region, 83.3% of the herds had experienced a diarrhea outbreak, of which 66.7% had happened more than nine months ago and 16.7% within the last three months (Additional file 2). In the W region, 25% of the herds had experienced a diarrhea outbreak, which both had happened more than six months ago. Regarding use of antimicrobial for treatment of diarrhea, 33.3% of the farmers in the NE region and 62.5% in the W region claimed to never or rarely use such treatment. Most farmers in the NE region (58.3%) but only 25% of the farmers in the W region answered that they sometimes treat calves with antimicrobials. A majority (66.6%) of the farmers in the NE region had not observed a difference in calf health between seasons, while the majority (62.5%) in the W region answered yes to seasonal variations, where winter was the hardest season for the calves. Half (50%) of the farmers in the NE region fed colostrum to the newborn calf within two hours, while only 25% in the W region did the same. The timing of first feeding was very different between farms in the W region, ranging from feeding within two hours to over six hours. No farmer used a colostrum bank or checked the colostrum quality regularly. Vaccination of cows against mastitis was carried out in 25% of the herds, all of which were in the NE region (Additional file 2).

Among the six herds that purchased calves from external herds, only two (one per region) practiced isolation prior to herd integration. When comparing herds that acquired calves from other herds to those that did not, a higher average number of both respiratory and enteric pathogens in the pools was observed in the former group.

## Discussion

The location of Iceland as a geographically isolated unit and the strict regulations on livestock importation make it interesting to investigate which pathogens are present among Icelandic calves especially because few data have been published about this. The sampled calves within this study were located at two different regions of the country, where the weather in the NE region is generally colder and has greater temperature fluctuations compared to the W region with milder and wetter weather due to influence of ocean currents. An interesting difference in the pathogen detection was that BCoV, *M. haemolytica* and *P. multocida* were not detected in any samples collected in the W region, whereas the other pathogens were detected in both regions. However, this difference cannot be explained solely by the apparent weather differences between the two regions. Furthermore, it cannot be ruled out that BCoV, *M. haemolytica*, and *P. multocida* might have been detected in herds in the W region if sampling had also been conducted in the spring, as in the NE region, since some of these pathogens may exhibit seasonal patterns. A notable contrast between the two regions was the incidence of diarrhea outbreaks reported by the farmers via the questionnaire (NE: 83% vs. W: 25%). Since these outbreaks had happened a while before the start of this study it was not possible to obtain any sample material from these outbreaks. It would have been interesting to investigate which pathogens were involved in these outbreaks.

Most Icelandic calves are housed indoors year-round, and all calves included in this study were housed indoors at the time of sampling, with most of them kept in group pens on either straw bedding or slatted floors. This indoor housing contrasts with practices in many other countries, where calves are often housed outdoors in calf hutches. This could partially explain why so little difference in the occurrence and presence of the different pathogens was observed between the two seasons (spring and fall) in the NE region. Furthermore, no clear differences in the occurrence or the severity of the clinical signs were observed between the two seasons. It would have been interesting to assess whether the differences observed between the spring and fall samplings observed in the region NE would have been similar if spring sampling had also been conducted in the W region. Other studies have found that nasal discharge and coughing occur more frequently during the winter months compared to summer [2, 25]. However, the influence of season on calf health can be difficult to compare between countries, as weather conditions vary significantly. Another difference compared with many cattle herds abroad is the number of animals per herd. Herds in Iceland are generally small, and smaller herd sizes often result in reduced infection pressure simply because fewer animals are available to become infected. Furthermore, herds that did not purchase animals from outside exhibited fewer pathogen detections compared with herds that introduced calves from other herds, consistent with the well-established role of animal movement as a major route for pathogen introduction [26].

Distribution of the respiratory pathogens in the three age groups across herds, regions and seasons showed that the occurrence of *T. pyogenes*, *P. multocida* and *H. somni* increased with age, while it decreased for *Mycoplasma* spp., and *M. haemolytica* was only detected in the oldest calves. Interestingly, even though these bacteria were present, very few clinical signs of respiratory disease in the sampled calves were observed. This could be explained by the fact that these bacteria are all natural commensals in calves, primarily inhabiting the upper respiratory tract. However, they all have the capacity to become opportunistic pathogens when host-related or environmental conditions become conducive, such as during periods of stress, transportation, immunosuppression or viral infection that is known to predispose the lungs to secondary bacterial infections [27, 28].

BCoV was the only BRD associated virus detected in the Icelandic calves. This virus is endemic in Iceland and has been associated with reduction in milk yield in several herds (personal communication, Vilhjálmur Svansson, Institute for Experimental Pathology at Keldur, University of Iceland). Besides its ability to cause respiratory illness and diarrhea in calves [29], BCoV is also associated with winter dysentery, which is a highly contagious, acute gastrointestinal disease in adult dairy cattle. This phenomenon is characterized by diarrhea, often bloody, and a dramatic drop in milk production [30, 31]. This study confirms the presence of BCoV in Icelandic calves, but no association with disease could be assessed because the nasal swab samples were tested in pools. Moreover, due to the low number of clinically affected animals and the small sample size, the uncertainty surrounding such estimates would be considerable.

BCoV and RVA were detected in around a quarter of the fecal pools, each showing a different detection pattern. BCoV was present in all three age groups, but only in the NE region, whereas RVA was detected in age groups 1 and 2 in both regions. RVA was detected with the highest occurrence in the youngest calves (age group 1, *P* = 0.001), which aligns with findings from previous studies [32, 33]. To our knowledge, detection of RVA has not previously been reported in Iceland.

According to the questionnaire, only five of the 20 herds had a vaccination strategy for the cows, which all were against mastitis. Vaccination of cows to minimize the occurrence of diarrhea in the calves is relatively common abroad. However, this is not very widespread in Iceland, as about it is not often known which pathogen(s) are causing the diarrhea in the individual herds. For immunization against both BCoV and RVA, vaccines are administered to pregnant cows or heifers to increase pathogen-specific maternal antibody levels, which are subsequently transferred to the newborn calf via colostrum. To ensure optimal passive immunity, it is essential that the calf receives high‑quality colostrum shortly after birth [34]. However, none of the farmers reported routinely assessing colostrum quality, which can result in insufficient immunization of the calves despite appropriate vaccination of the dams. Vaccination against RVA can somehow be more complicated since several genotypes have been described to be present in calves [32, 35]. Therefore, it would be relevant to further investigate which genotypes of RVA are present in the Icelandic calves.

## Conclusions

Several of the investigated pathogens were shown to be present in Icelandic calves, with some of them being described for the first time in Iceland. Three of the pathogens (BCoV, *M. haemolytica*, and *P. multocida*) were only detected in the NE region, but no pronounced differences in pathogen circulation among the studied herds in the NE region in the spring and fall were found. Diarrhea was the most widespread clinical sign recorded among the calves, and half of the diarrheic calves tested positive for RVA, with the majority of these originating from the youngest age group. These findings improve the understanding of pathogen distribution in Icelandic calves, and knowledge of the pathogens present within a herd or calf population may support more targeted treatment approaches and informed vaccination decisions.

## Supplementary Information

Supplementary Material 1. Herd number, herd location, sample collection date, calf number, age group and clinical score. Clinical registrations for the 197 examined calves from the 20 herds located either in the Northeastern (NE) or Western (W) region of Iceland

Supplementary Material 2. Questionnaire regarding biosecurity, calf health, colostrum management and other management practices. Questionnaire responses from the participating farmers

## Data Availability

The datasets used and/or analyzed during the current study are available from the corresponding author on reasonable request.

## Abbreviations

BCoV: Bovine coronavirus
BHV-1: Bovine herpesvirus 1
BPiV3: Bovine parainfluenza virus type 3
BRD: Bovine respiratory disease
BRSV: Bovine respiratory syncytial virus
BVDV: Bovine viral diarrhea virus
IDV: Influenza D virus
DA: Dynamic array
GLMM: Generalized linear mixed model
IFC: Integrated fluidic circuit
NE: Northeastern
qPCR: Quantitative real-time PCR
RVA: Rotavirus A
Spp: Species
W: Western
